# Slow Protein Turnover Explains Limited Protein-Level Response to Diurnal Transcriptional Oscillations in Cyanobacteria

**DOI:** 10.3389/fmicb.2021.657379

**Published:** 2021-04-14

**Authors:** Jan Karlsen, Johannes Asplund-Samuelsson, Michael Jahn, Dóra Vitay, Elton P. Hudson

**Affiliations:** ^1^Department of Protein Science, KTH Royal Institute of Technology, Stockholm, Sweden; ^2^Science for Life Laboratory, Stockholm, Sweden; ^3^Biosyntia ApS, Copenhagen, Denmark

**Keywords:** cyanobacteria, diurnal gene expression, protein turnover, post-transcriptional regulation, metabolic regulation, RNA sequencing, ribosome profiling, proteomics

## Abstract

Metabolically engineered cyanobacteria have the potential to mitigate anthropogenic CO_2_ emissions by converting CO_2_ into renewable fuels and chemicals. Yet, better understanding of metabolic regulation in cyanobacteria is required to develop more productive strains that can make industrial scale-up economically feasible. The aim of this study was to find the cause for the previously reported inconsistency between oscillating transcription and constant protein levels under day-night growth conditions. To determine whether translational regulation counteracts transcriptional changes, *Synechocystis* sp. PCC 6803 was cultivated in an artificial day-night setting and the level of transcription, translation and protein was measured across the genome at different time points using mRNA sequencing, ribosome profiling and quantitative proteomics. Furthermore, the effect of protein turnover on the amplitude of protein oscillations was investigated through *in silico* simulations using a protein mass balance model. Our experimental analysis revealed that protein oscillations were not dampened by translational regulation, as evidenced by high correlation between translational and transcriptional oscillations (*r* = 0.88) and unchanged protein levels. Instead, model simulations showed that these observations can be attributed to a slow protein turnover, which reduces the effect of protein synthesis oscillations on the protein level. In conclusion, these results suggest that cyanobacteria have evolved to govern diurnal metabolic shifts through allosteric regulatory mechanisms in order to avoid the energy burden of replacing the proteome on a daily basis. Identification and manipulation of such mechanisms could be part of a metabolic engineering strategy for overproduction of chemicals.

## Introduction

Knowledge of cyanobacterial metabolism and its regulation can guide metabolic engineering efforts to create more efficient strains for renewable fuel and chemical production. As their energy source is limited to the light hours of the day, cyanobacteria have evolved to shift between photosynthetic and respiratory metabolism between day and night, respectively. During the day, CO_2_ is fixed in the Calvin cycle and converted into biomass, including storage compounds such as glycogen. During the night, CO_2_ fixation and most biosynthetic pathways are inactive while glycogen is degraded to support cellular maintenance and a small subset of pathways that prepare the cell for the next light period (Saha et al., 2016; Reimers et al., 2017; Welkie et al., 2019; Werner et al., 2019). Metabolic shifts that occur at specific time points over the day-night cycle are governed by regulating the flux through key enzymes and pathways. The flux through an enzyme is regulated by changing its abundance, product/substrate concentration, or through post-translational effects that alter its apparent kinetic parameters. Several studies have investigated abundance-controlled regulation by tracking changes in the cyanobacterial transcriptome and proteome across the day-night cycle. Transcriptomic data collected from a range of cyanobacteria showed that a large fraction of cyanobacterial transcripts oscillates diurnally (30–87%), with peak expression mostly during the transitions between day and night (Stöckel et al., 2008; Ito et al., 2009; Waldbauer et al., 2012; Saha et al., 2016). Additionally, many transcripts tend to peak just before the time when the gene product’s function is expected to be needed by the cell. For example, transcripts of Calvin cycle and pentose phosphate pathway genes peaked in the beginning of the light and dark period, respectively (Waldbauer et al., 2012). Yet surprisingly, a few proteomics studies have shown that abundance of most proteins remains nearly constant (Stöckel et al., 2011; Waldbauer et al., 2012; Guerreiro et al., 2014; Angermayr et al., 2016). This makes the regulatory purpose of time-dependent transcription seem insignificant for regulating enzyme activity and diurnal metabolic shifts.

The underlying cause for a broad discrepancy between transcript and protein dynamics is still not clear, but it could be attributed to post-transcriptional regulation or low daily *de novo* protein synthesis relative to the protein abundance. One possibility is that translational regulation counteracts changes in mRNA abundance, resulting in reduced variation in protein synthesis rate of genes despite their altered transcript levels. Protein synthesis rates can be measured genome-wide through ribosome profiling (Ribo-Seq), which quantifies the total number of ribosomes translating a gene’s transcripts (Brar and Weissman, 2015; Liu et al., 2017). A translationally-regulated gene would show a change in ribosome abundance that is not equal to the change in transcript abundance, or vice versa. Translational regulation was shown to occur in 7% of the genome of *Synechocystis* sp. PCC 6803 (*Synechocystis*) in response to CO_2_ starvation (Karlsen et al., 2018). A second possibility is that protein levels are held relatively constant by active protein degradation. However, rapid degradation of newly synthesized proteins would waste energy and cellular resources and reduce fitness. Lastly, relatively low variation in protein levels could also occur without any post-transcriptional regulation, if the daily variation in protein synthesis rate is low compared to the protein abundance, i.e., if the turnover rate of the proteome is low.

Here, we apply a systems biology approach to take a closer look at the discrepancies between transcription and protein abundances during day-night cycles in cyanobacteria. The model cyanobacterium *Synechocystis* was grown in controlled turbidostat cultures under artificial day-night cycles. To assess the impact of translational regulation on the protein level, the transcriptome, translatome, and proteome was measured at different time points using mRNA sequencing, ribosome profiling, and quantitative proteomics. We found that protein synthesis rates tracked with transcriptional oscillations, while protein abundances remained relatively constant, indicating that translational regulation does not significantly impact the protein-level behavior. We further investigated the effect of protein turnover on protein dynamics *in silico*. Simulation of protein oscillations using biologically relevant parameter settings, resulted in a protein amplitude similar to experimental observations. The data and model simulations demonstrate that post-translational regulation is not necessary for the proteome to remain stable, even under significant transcriptional oscillations.

## Materials and Methods

### Cultivation and Sampling

*Synechocystis* sp. PCC 6803 was cultivated in 1.6 L BG-11 (pH = 7.8) at 30°C in a cylindrical photobioreactor (D = 10 cm, V = 2 L, baffled). The culture was illuminated with an LED light jacket covering the sides of the cylinder (90% red light, 10% blue light). CO_2_-enriched air was sparged into the culture (7% CO_2_, 330 mL min^–1^) and the impeller stirring rate was set to 150 rpm. Cells were grown in turbidostat mode (OD_730_ set point: 0.65–0.80) under an artificial day-night light regime (Day: sinusoidal, max 500 μmol photons m^–2^ s^–1^; Night: dark) for seven days, at which point the diurnal pattern of dissolved oxygen (growth rate proxy) became stable over subsequent days. Five time points (1 h before/after sunrise, midday, 1 h before/after sunset) were then sampled at -1, 1, 6, 11, 13, 30, 35, 37, 47, and 49 h relative to the first subjected sunrise. Two replicate cultivations were conducted. In the first cultivation, two replicate samples were collected at all five diurnal time points for mRNA sequencing and ribosome profiling. In the second cultivation, one and two replicate samples were collected for ribosome profiling and quantitative proteomics, respectively. Eleven out of fifteen collected ribosome profiling samples were analyzed, which resulted in two replicate measurements at all time points, except 1 h after subjected sunrise which had three. The correlation between ribosome profiling replicates within the same cultivation was similar to the correlation between replicates of different cultivations (*r* ≈ 0.99 and *r* ≈ 0.97, respectively), which indicated that results were reproducible across cultivations (Supplementary Figure 1). Cultivation data is shown in Supplementary Figure 2.

### Determination of Diurnal Growth Rate

Analysis was performed using R v.3.6 scripts^1^. Specific growth rates (μ) were determined over the time course of the first cultivation at 30-second intervals according to a mass balance-derived equation of biomass in the culture:

$$\mu =\left(\frac{d\,O\,D_{730}}{d\,t}+D\cdot O\,D_{730}\right)/O\,D_{730}.$$

Where D is the dilution rate (h^–1^) and OD_730_ is the optical density (cell mass concentration proxy) of the culture. Dilution rates were calculated by dividing time-specific medium feed rates (L h^–1^, automatically regulated) with the volume of the culture (1.6 L). To remove noise, each time point was assigned with the 40% truncated average of the closest 480 time points (40% most extreme values removed in each window). The noise filtering step was repeated once. The optical density was measured automatically at 880 nm and converted to OD_730_ using a conversion factor based on offline OD_730_ measurements during the time course. Noise was removed from OD_730_ values in two subsequent filtration steps. In the first step, each time point was assigned with the 40% truncated average of the 960 closest time points. In the second step, each time point was assigned with the average of the closest 360 time points. The change in OD_730_ over time ($\frac{d\,O\,D_{730}}{d\,t}$) was calculated at each time point as the slope in noise-removed OD_730_ (1 h centered time intervals). Noise was finally removed from calculated growth rates by assigning each time point with the 40% truncated average of the 720 closest time points, and negative values were replaced by zero. The average diurnal growth rate was based on growth rates determined across the two days of sampling (0–48 h). Growth rates determined in the second cultivation experiment were prone to error and therefore not reported (higher noise levels and longer data acquisition intervals). Data is shown in Supplementary Figure 2.

### Sample Preparation for mRNA Sequencing

Culture medium was removed by centrifugation and cell pellets snap-frozen in liquid nitrogen (stored at –80°C). Cell lysis was performed using lysozyme treatment and vortexing with glass beads. Total RNA was extracted from the cleared lysate with hot phenol/chloroform and isopropanol precipitation and remaining DNA was removed using DNase I. The amount of rRNA was subsequently reduced using the *RiboMinus Kit, Bacteria* (*ThermoFisher, K155004*) according to the manufacturer’s instructions. Sequencing libraries were prepared using the *NEBNext Ultra Directional RNA Library Prep Kit* (*New England Biolabs, E7420*). Libraries were sequenced on an Illumina NextSeq500 platform (75 bp read length, single end). For details, see Karlsen et al. (2018). Raw sequencing data are available at the European Nucleotide Archive under accession number PRJEB42778.

### Sample Preparation for Ribosome Profiling

Cells were rapidly harvested by vacuum filtration and snap-frozen in liquid nitrogen (stored at –80°C). Frozen cells were lysed using cryogenic grinding. The frozen lysate was thawed and cell debris was removed by centrifugation. Polysomes in the lysate were immediately digested with micrococcal nuclease, and generated monosomes were extracted by sucrose gradient ultracentrifugation and fractionation. Total RNA was extracted from monosomes with hot phenol/chloroform and isopropanol precipitation. Ribosome protected mRNA fragments were then extracted by size selection on a denaturing polyacrylamide gel (20–40 nt) and subsequently converted into a sequencing library using the *NEBNext small RNA library prep set (New England Biolabs; E7300)*. Libraries were sequenced on an Illumina NextSeq500 platform (75 bp read length, single end). For details, see Karlsen et al. (2018). Raw sequencing data are available at the European Nucleotide Archive under accession number PRJEB42778.

### Sample Preparation for LC-MS-MS

Culture medium was removed by centrifugation and cell pellets snap-frozen in liquid nitrogen (stored at –80°C). Thawed cell pellets were suspended in 125 μL solubilization buffer (200 mM TEAB, 8 M Urea, protease inhibitor). 100 μL glass beads (100 μm diameter) were added to the cell suspension and cells were lysed by bead beating in a Qiagen TissueLyzer II (5 min, f = 30/s, precooled cassettes). Cell debris was removed by centrifugation at 14,000 × *g*, 30 min, 4°C, and supernatant was transferred to a new tube. Protein concentration was determined using the Bradford assay (Bio-Rad). For reduction and alkylation of proteins, 2.5 μL 200 mM DTT (5 mM final) and 5 μL 200 mM CAA (10 mM final) were added, respectively, and samples incubated for 60 min at RT in the dark. Samples were diluted 8-fold with 700 μL 200 μM TEAB. For digestion, Lys-C was added in a ratio of 1:75 w/w to protein concentration, and samples were incubated at 37°C and 600 RPM for 12 h. Trypsin was added (1:75 w/w) and samples incubated for 24 h at the same conditions. Samples were acidified with 100 μL 10% formic acid (FA) and insoluble compounds were removed by centrifugation (14,000 × *g*, 15 min, RT). Peptide samples were then cleaned up using a solid phase extraction protocol (Sep-Pak 1cc 50 mg A C18 cartridges, Waters) according to the manufacturer’s recommendations. Briefly, Sep-Pak columns were equilibrated with 1 mL acetonitrile (ACN) and 2 × 1 mL 0.6% acetic acid. Samples were loaded on columns and washed twice with 1 mL 0.6% acetic acid. Peptides were eluted from the column in 500 μL elution buffer (0.6% acetic acid, 80% ACN) and dried in a speedvac for 2 h, 37°C. Dried peptides were frozen at –80°C and dissolved in 10% FA to a final concentration of 1 μg/μL before MS measurement.

### LC-MS-MS Analysis of Lysates

Lysates were analyzed using a Thermo Fisher Q Exactive HF mass spectrometer (MS) coupled to a Dionex UltiMate 3000 UHPLC system (Thermo Fisher). The UHPLC was equipped with a trap column (Acclaim PepMap 100, 75 μm × 2 cm, C18, P/N 164535, Thermo Fisher Scientific) and a 50 cm analytical column (Acclaim PepMap 100, 75 μm × 50 cm, C18, P/N ES803, Thermo Fisher Scientific). The injection volume was 2 μL out of 18 μL in which the samples were dissolved in the autosampler. Chromatography was performed using solvent A (3% ACN, 0.1% FA) and solvent B (95% ACN, 0.1% FA) as the mobile phases. The peptides were eluted from the UHPLC system over a 90 min gradient at a flow rate of 250 nL/min with the following mobile phase gradient: 2% solvent B for 4 min, 2–4% solvent B for 1 min, 4–45% solvent B for 90 min, 45–99% solvent B for 3 min, 99% solvent B for 10 min and 99–2% solvent B for 1 min following re-equilibration of the column at 2% solvent B for 6 min. The MS was operated in a data-dependent acquisition mode with a Top 8 method. The MS was configured to perform a survey scan from 300 to 2,000 m/z with resolution of 120,000, AGC target of 1 × 10^6^, maximum IT of 250 ms and 8 subsequent MS/MS scans at 30,000 resolution with isolation window of 2.0 m/z, AGC target of 2 × 10^5^, maximum IT 150 ms and dynamic exclusion set to 20 s. LC-MS shotgun proteomics data are available at the PRIDE Archive^2^ under accession number PXD023812.

### Relative Quantification of Cellular Protein Content

The protein content was quantified in the cell extracts used for LC-MS-MS (Bradford assay). Measured concentrations were normalized to the sample’s cell mass concentration (based on external OD_730_ measurements).

### Sequencing Data Processing and Quantification of mRNA and Ribosomes

Analysis of sequencing data was conducted using python v.2.7 scripts adapted from Becker et al. (2013), R v.3.4 scripts, and bash commands parallelized using GNU Parallel v.20161222 (Tange, 2011)^3^. FastQC was used to assess the quality and general features of sequencing datasets (Andrews, 2010). Adapter sequences were trimmed off using Cutadapt v1.18 (Martin, 2011). Base calls with a Sanger quality score lower than 20 were trimmed off the ends of mRNA sequencing reads using Sickle (Joshi and Fass, 2011). Ribosome profiling reads with an average Sanger quality score lower than 25 were removed using Seqmagick v0.6.2^4^. Reads shorter than 6 nt were discarded. Reads that mapped to tRNA and rRNA genome sequences were subsequently removed using Bowtie v.1.2.2 (Langmead et al., 2009). Bowtie was used to map remaining reads to the genome, including plasmids (NC_000911.1 + NC_005229.1 + NC_005230.1 + NC_005231.1 + NC_005232.1 + NC_020289.1 + NC_020290.1 + NC_020298.1). A maximum of two alignment mismatches were allowed. If a read mapped to several locations, only the one best alignment was kept. The read was discarded if it could not be mapped to a unique location in this way. The total number of mapped non-tRNA/rRNA reads was ∼2 million and 34–78 million in mRNA sequencing and ribosome profiling samples, respectively. For each mapped mRNA sequencing read, a read count equal to 1 was distributed evenly over all its aligned genome positions. In contrast, the read count of each mapped ribosome profiling read was assigned to a single genome position, 12 nt upstream of the aligned 3’ end. This assigns the read count to the genome position covered by the A-site of the ribosome (Karlsen et al., 2018). As only ribosome profiling reads longer than 24 nt were counted, the total number of counted mapped reads per sample was between 17 and 68 million. The mRNA/ribosome abundance of a gene (RPKM) was quantified by dividing the read count on the gene’s coding sequence with the length of the coding sequence (in 1,000 base pairs) and the total number of counted reads on all coding sequences (in million). Coding sequences were defined according to GenBank files for the NCBI reference sequences NC_000911.1, NC_005229.1, NC_005230.1, NC_005231.1, NC_005232.1, NC_020289.1, NC_020290.1, and NC_020298.1.

### Protein Identification and Quantification

Thermo raw spectra files were converted to the mzML standard using Proteowizard’s MSConvert tool (Adusumilli and Mallick, 2017). Peptide identification and label-free quantification were performed using OpenMS 2.4.0 in KNIME (Röst et al., 2016). The KNIME pipeline for MS data processing was deposited on https://github.com/m-jahn/openMS-workflows (labelfree_MSGFplus_Percolator_FFI.knwf). MS/MS spectra were subjected to sequence database searching using the OpenMS implementation of MS-GF+ (Granholm et al., 2014) with the *Synechocystis* sp. PCC 6803 reference proteome as database (as of 04 April 2019). Carbamidomethylation was considered as a fixed modification on cysteine and oxidation as a variable modification on methionine. The precursor ion mass window tolerance was set to 10 ppm. The *PeptideIndexer* module was used to annotate peptide hits with their corresponding target or decoy status, *PSMFeatureExtractor* was used to annotate additional characteristics to features, *PercolatorAdapter* was used to estimate the false discovery rate (FDR), and *IDFilter* was used to keep only peptides with q-values lower than 0.01 (1% FDR). The quantification pipeline is based on the *FeatureFinderIdentification* workflow allowing feature propagation between different runs (Weisser and Choudhary, 2017). MzML files were retention time corrected using *MapRTTransformer*, and identifications (idXML files) were combined using the *IDMerger* module. *FeatureFinderIdentification* was then used to generate featureXML files based on all identifications combined from different runs. Individual feature maps were combined to a consensus feature map using *FeatureLinkerUnlabelledKD*, and global intensity was normalized using *ConsensusMapNormalizer* (by median). Protein quantity was determined by summing up the intensities of all unique peptides per protein using *ProteinQuantifier*.

### Integrated Analysis of Diurnal mRNA, Ribosome and Protein Oscillations

Analysis was performed using R v.3.6 scripts^1^. Genes with less than 30 and 60 reads were initially removed from the mRNA sequencing dataset and ribosome profiling dataset, respectively. In the proteomics dataset, inaccurately measured proteins were removed by discarding those with a log_2_ intensity standard deviation greater than 1 at any time point. Only genes with at least two replicate measurements across all time points and across all three datasets were analyzed. The total number of genes (*n* = 1,126) was mainly limited by the proteomics dataset. Abundance values for each gene were log_2_ transformed and then centered around the gene’s daily average log_2_ abundance to reflect relative fold changes. To identify genes with diurnal changes in mRNA abundance (considered “cyclic”), differential abundance between time points was analyzed with one-way ANOVA. A gene’s mRNA abundance was considered to change significantly over the day-night cycle if (1) the Benjamini-Hochberg adjusted q-value was less than 0.1, and if (2) the absolute log_2_ fold change was greater than 1, between any two time points. Cyclic genes were then clustered into four groups (G1–G4) according to their diurnal mRNA abundance pattern using hierarchical clustering (R function: *hcluster*; distance measure: Pearson correlation; linkage method: Ward). Maximum cluster separation was obtained when choosing a cluster number of 2 and 4 (average silhouette width of 0.63 and 0.49, respectively). Four clusters were chosen as relatively unique diurnal patterns were visible in each, despite lower average silhouette width. Non-cyclic genes were assigned to a fifth group (G0). The peak-to-peak relative amplitude in mRNA, ribosome and protein abundance was calculated as the maximum log_2_ fold change between sample mean values across the day-night cycle. The median relative amplitude of cyclic genes in each dataset was used to summarize and compare the overall relative amplitude observed at transcriptional, translational and protein level. Differential log_2_ protein abundance across time points was assessed for cyclic and non-cyclic genes using one-way ANOVA. A Benjamini-Hochberg adjusted q-value less than 0.1 was considered significant.

### Modeling of Protein Oscillations

The change over time of an arbitrary gene’s (J) protein concentration (P_*J*_) was expressed according to the cellular mass balance of that protein:

(1) $$\frac{d\,P_{J}}{d\,t}=F_{S,J}\cdot S_{T\,O\,T}-F_{P,J}\cdot P_{T\,O\,T}\cdot \mu -F_{P,J}\cdot P_{T\,O\,T}\cdot k_{D,J}$$

Where F_*S, J*_ is the fraction of total bulk protein synthesis (S_*TOT*_) dedicated to protein J, F_*P, J*_ is the fraction of the total cellular protein concentration (P_*TOT*_) made up by protein J, μ is the growth rate, and k_*D, J*_ is the gene-specific degradation rate of protein J.

In a similar manner, the rate change of P_*TOT*_ was expressed according to the cellular mass balance of total protein:

(2) $$\frac{d\,P_{T\,O\,T}}{d\,t}=S_{T\,O\,T}-P_{T\,O\,T}\cdot \mu -P_{T\,O\,T}\cdot k_{D,M\,E\,A\,N}$$

Where k_*D, MEAN*_ is the bulk protein degradation rate.

Under the assumption of a constant P_*TOT*_, the term S_*TOT*_ is constrained to be proportional to the sum of μ and k_*D, MEAN*_:

(3) $$S_{T\,O\,T}=P_{T\,O\,T}\cdot \left(\mu +k_{D,M\,E\,A\,N}\right)$$

Substitution of Eq. 3 into Eq. 1 yields:

(4) $$\frac{d\,P_{J}}{d\,t}=P_{T\,O\,T}\cdot \left(F_{S,J}\cdot \left(\mu +k_{D,M\,E\,A\,N}\right)-F_{P,J}\cdot \left(\mu +k_{D,J}\right)\right)$$

For the model to better reflect the abundance fraction of protein J measured by protein mass spectrometry, the expression *P*_*J*_ = *F*_*P*,*J*_⋅*P*_*TOT*_ as substituted into Eq. 4:

(5) $$\frac{d\,F_{P,J}}{d\,t}=F_{S,J}\cdot \left(\mu +k_{D,M\,E\,A\,N}\right)-F_{P,J}\cdot \left(\mu +k_{D,J}\right)$$

Considering an “average” protein J for which k_*D, J*_ = k_*D, MEAN*_, Eq. 5 can be further simplified to:

(6) $$\frac{d\,F_{P,J}}{d\,t}=\left(\mu +k_{D,M\,E\,A\,N}\right)\cdot \left(F_{P,J}-F_{S,J}\right)$$

Simulations of diurnal protein abundance oscillations were performed using R v.3.6 scripts^1^. The model equation (Eq. 5) was solved numerically using an ordinary differential equation solver (R function: *ode*). F_*S,J*_ was set as a function of time with an amplitude fold change corresponding to the experimentally observed median value:

$$F_{S,J}=\left(3.05-1\right)/\left(3.05+1\right)\cdot s\,i\,n\,\left(2\cdot \pi /24\cdot t+0.5\,\pi \right)+1.$$

The behavior of F_*P, J*_ over time in response to different protein turnover scenarios was analyzed by altering the settings of the remaining input parameters μ, k_*D, MEAN*_ and k_*D, J*_. To simulate anticorrelated degradation vs. synthesis, k_*D, J*_ was expressed as a sine function with a 12 h phase shift relative to F_*S,J*_:

$$k_{D,J}=0.1\cdot s\,i\,n\,\left(2\cdot \pi /24\cdot t+1.5\,\pi \right)+0.1.$$

A time depended growth rate was modeled by expressing μ as a sine function during day time:

μ=0.05⋅s⁢i⁢n⁢(2⋅π/24⋅t)

and as zero during night time.

## Results

### Diurnal Transcriptional Oscillations Are Not Dampened by Translation but Protein Levels Are Largely Constant

To investigate whether translational regulation causes protein levels to remain constant during day-night cycles in cyanobacteria, we performed genome-wide measurements of the transcriptome, translatome, and proteome in *Synechocystis* using mRNA sequencing, ribosome profiling, and quantitative shotgun proteomics, respectively. Cells were adapted to an artificial day-night regime for seven days in a controlled turbidostat culture and samples were collected over separate days at five time points: 1 h before and after artificial sunrise, midday, and 1 h before and after sunset. The maximum and average growth rate was 0.05 and 0.018 h^–1^, respectively, and correlated with the light intensity curve (Supplementary Figure 2).

**FIGURE 1:**
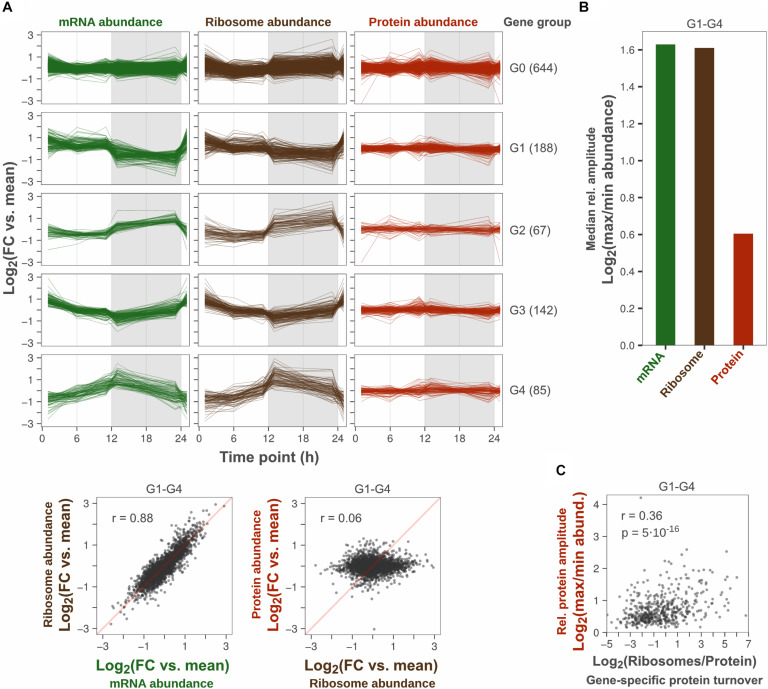
Protein amplitudes over a diurnal cycle are small relative to amplitudes in transcription and translation. **(A)** Comparison of diurnal expression patterns at the level of transcription, translation and protein. The log_2_-transformed fold change (FC) vs. the daily average gene-specific abundance of mRNA, ribosome and protein was plotted for each gene detected across all three levels (top). Time point 1 was plotted twice to visualize changes at sunrise. Genes were grouped according to their daily mRNA abundance pattern: No significant change (G0), Cyclic (G1–G4). The number of genes in each group is shown in parentheses. Scatter plots show the correlation of diurnal abundance patterns between levels among cyclic genes (bottom). Insets show the Pearson correlation coefficient. Duplicate measurements were performed at all time points, except 1 h after sunrise where ribosome abundance was measured in triplicates. **(B)** Comparison of the median peak-to-peak relative amplitude among cyclic genes. **(C)** Correlation of relative protein amplitude vs. protein synthesis to protein abundance ratio (inferred gene-specific protein turnover) among cyclic genes. The protein synthesis to protein abundance ratio was based on the daily average abundance of ribosomes and protein. Insets show the Pearson correlation coefficient (*r*) and the statistical significance of the trend (*p*-value).

A total of 1126 genes were analyzed, which had at least two replicate measurements across all time points in all three datasets. Of these, 43% showed cyclic diurnal mRNA expression (|log_2_FC| > 1, FDR < 0.1, Supplementary Table 1) which is in agreement with microarray-based transcriptomics studies from *Synechocystis* and *Synechococcus elongatus* PCC 7942 grown in diurnal light conditions (Guerreiro et al., 2014; Saha et al., 2016). These genes were designated “cyclic genes” and clustered according to their diurnal mRNA abundance pattern into four groups: “G1-G4.” Non-cyclic genes were assigned to a fifth group: “G0” (Figure 1A). Protein synthesis rates, inferred from the number of translating ribosomes, correlated well with cyclic transcription patterns (*r* = 0.88, Figure 1A). This implies a low degree of translational regulation and confirms that protein synthesis rates oscillate significantly over the day night cycle, in concert with transcript levels. In contrast, oscillation patterns could not be distinguished at the protein level which remained relatively constant. Since the variance between time points was low relative to the variance between replicates, no significant change in protein abundance was found across any time point (ANOVA, FDR < 0.1, Supplementary Table 1). This further explains the low correlation (*r* = 0.06) between protein synthesis and protein abundance patterns in this dataset (Figure 1A).

To analyze the peak-to-peak oscillation amplitude at the level of transcription, translation and protein, the log_2_ fold change between the minimum and maximum time point mean abundance of mRNA, translating ribosome, and protein was calculated for each cyclic gene (termed “relative amplitude”). The median relative amplitude across genes was compared to quantify the overall amplitude reduction from protein synthesis to protein abundance (Figure 1B). The median amplitude was two times lower at level of protein (1.5-fold) compared to the level of transcription and translation (3.0-fold), which is similar to the 2.3-fold median ratio between transcript and protein oscillations reported by Waldbauer et al. (2012). However, the median protein amplitude was probably overestimated here since the error of time point means was high relative to the variation between time point means. For example, if the error of time point means are large and the true protein amplitude is small for a gene, the measured variation across time points will be mostly noise. Consequently, the calculated relative amplitude is likely to be mostly noise, as it will be determined from the maximum fold change across five error-prone time point means. The variance between replicates was larger than the variance between time points for 41% of cyclic genes (ANOVA, SSB/SSW > 1). Thus, the determined median relative protein amplitude is likely to provide a certain over-estimated relative amplitude. Even though gene-specific protein amplitudes could not be determined with precision, there was a trend that proteins with high turnover rate (daily mean protein synthesis rate/daily mean protein abundance) had stronger oscillations (Figure 1C).

In conclusion, our multi-omics analysis shows that the decrease in oscillation amplitude between the mRNA level and the protein level is not caused by translational regulation of protein synthesis. The ratio between the median mRNA oscillation amplitude and the median protein oscillation amplitude was estimated to be more than twofold.

### A Slow Protein Turnover Reduces the Amplitude of Protein Abundance Oscillations

Protein concentrations in cyanobacteria remain largely constant over diurnal cycles, despite significant fluctuations in transcription and protein synthesis. A possible and intuitive explanation is that diurnal peaks in synthesis are counteracted by increased protein degradation. At the same time, this seems unlikely in an evolutionary context, as it implies an ineffective use of cellular resources which would result in decreased fitness. Therefore, we sought to determine whether this observation could solely be the result of a slow protein turnover. For this purpose, we applied a mass balance-based model that describes the change in an arbitrary protein’s concentration in response to diurnal synthesis oscillations (see section “Materials and Methods”). The model takes into account the synthesis, degradation, and growth dilution of the modeled protein (protein J) as well as the synthesis, degradation, and growth dilution of the bulk proteome. The model assumes that the total cellular protein concentration is constant, which has been shown experimentally in cyanobacteria over a range of growth rates and genetic perturbations (Touloupakis et al., 2015; Zheng and O’Shea, 2017). This assumption constrains bulk protein synthesis to be proportional to protein depletion, which is the sum of two processes, bulk protein degradation (described by k_*D, MEAN*_) and dilution by cell growth (described by μ). Bulk protein turnover, defined as bulk protein synthesis rate divided by bulk protein abundance, is thus also proportional to protein depletion (see section “Materials and Methods,” Eq. 3). We measured the total protein concentration in cell extracts and found it to be constant across time points (*p* ≥ 0.4, Supplementary Figure 3). In any case, deviations from this assumption do not have a significant impact on the amplitude of protein oscillations as long as the change is restored within the time span of the day-night cycle (see section “Discussion”).

**FIGURE 2:**
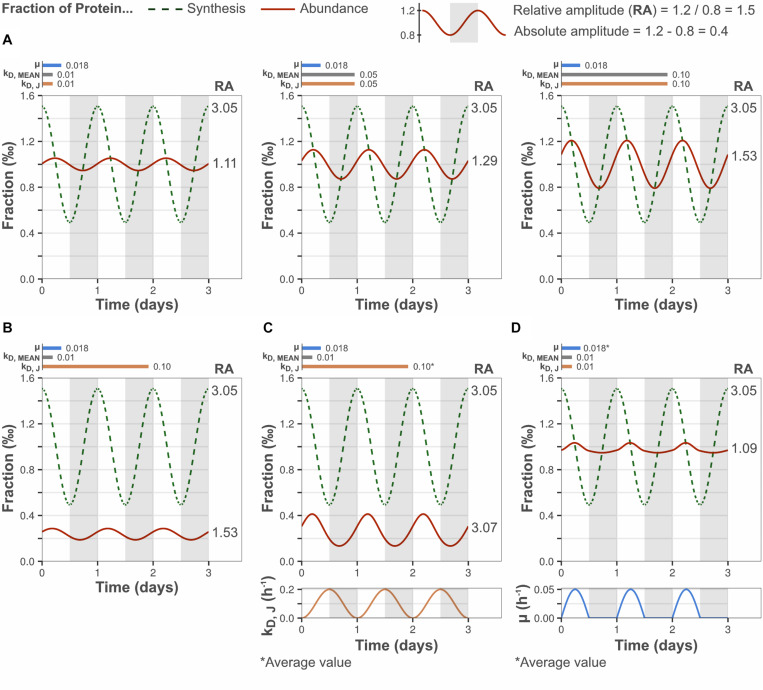
Low protein turnover reduces the relative amplitude of diurnal protein oscillations. Y-axes display the fraction of bulk protein synthesis (dashed lines) and the fraction of bulk protein abundance (solid lines) taken up by a modeled gene J. Bar plots show the selected growth rate (μ), bulk protein degradation rate (k_*D, MEAN*_) and gene-specific protein degradation rate (k_*D, J*_). **(A)** Higher bulk protein turnover (μ + k_*D, MEAN*_) increased the relative amplitude (RA) of diurnal protein oscillations, by increasing the absolute amplitude. k_*D, J*_ was set equal to k_*D, MEAN*_ to simulate the behavior of an “average” gene. **(B)** Increasing the gene-specific turnover, by setting the gene-specific protein degradation rate greater than bulk protein degradation rate (k_*D, J*_ > k_*D, MEAN*_; gene-specific turnover > bulk turnover), increased the relative protein amplitude by decreasing the daily average protein abundance. However, the absolute protein amplitude was not affected (compare **A**, left panel). **(C)** A high and fluctuating gene-specific protein degradation that is anticorrelated to the protein synthesis rate increased the relative protein amplitude by increased absolute protein amplitude, and by reduced daily average protein abundance. **(D)** A diurnally fluctuating growth rate had no significant effect on the protein amplitude, although the diurnal pattern was altered. The daily average growth rate was equal to the set value in Figure 1A. The growth rate curve was based on the experimentally determined pattern.

The effect of protein turnover rate on diurnal protein oscillations was explored using the mathematical model described above. Model parameters were selected to simulate biologically relevant cellular scenarios. The synthesis rate of protein J was set to oscillate with an amplitude equal to the median amplitude determined by ribosome profiling (Figure 1B) and μ was set to the observed daily average (Supplementary Figure 2). The bulk protein degradation rate was set to the median degradation rate reported for microalgae and plants (0.01 h^–1^), as it has not been determined experimentally in cyanobacteria (Table 1). In a first simulation, the gene-specific degradation rate of protein J (k_*D, J*_) was set equal to the bulk degradation rate so as to mimic the response of an “average gene.” This resulted in a relative protein amplitude of 1.11-fold, which is similar to the relative protein amplitude determined from the experimental data (Figure 2A). Increasing the bulk turnover rate (μ + k_*D, MEAN*_) from 0.028 to 0.118 h^–1^ (by increasing k_*D, MEAN*_ from 0.01 to 0.1 h^–1^) resulted in increased relative amplitude (1.11–1.53-fold) and decreased lag time of a protein J’s oscillations. The change in relative amplitude was in this case caused by changes in the absolute protein abundance difference between peak and trough (termed “absolute amplitude,” see legend Figure 2). A positive correlation between protein amplitude and bulk protein turnover was also predicted implicitly in model Eq. 6, where a higher bulk turnover increases the protein response dP/dt, which leads to a faster change and increased amplitude (see section “Materials and Methods”). Equation 6 further shows that the direction of the protein change is determined by the difference in protein synthesis fraction and protein abundance fraction (F_*S, J*_–F_*P, J*_). The abundance fraction will therefore become equal to the synthesis fraction over time, if the synthesis rate of a gene J is constant (e.g., at steady state growth) and k_*D, J*_ = k_*D, MEAN*_. More importantly, this implies that a change in synthesis rate from one steady state to a new one, will result in an abundance change that is at most equal to the synthesis change, if given enough time to reach the new steady state (∼5 protein half-lives). Thus, under a diurnally changing synthesis rate, the protein amplitude is bound to be less than (or at most equal to) the protein synthesis amplitude, unless the protein half-life is much shorter than the time period of the day-night cycle (i.e., relatively high protein turnover).

**TABLE 1:** Reported median protein degradation rates and growth rates in different organisms.

Organism	Median protein deg. rate (k_D, MEAN_, h^–1^)	Growth rate (μ, h^–1^)
*Chlamydomonas reinhardtii* (Algae)	0.015^a^	0.011^a^
*Arabidopsis thaliana* (Plant)	0.010^b^ 0.0092^c^	0.0097^*b*^ 0.0013–0.0063^c^
*Lactococcus lactis* (Heterotrophic bacteria)	0.12–0.91^d^	0.1–0.5^d^
*Saccharomyces Cerevisiae* (Budding yeast)	0.97^e^	0.46^e^
Human (HeLa cells)	0.02^f^* 0.034^g^*	0.03^h^*

^*a*^Mastrobuoni et al. (2012); ^*b*^Li et al. (2012); ^*c*^Li et al. (2017); ^*d*^Lahtvee et al. (2014); ^*e*^Belle et al. (2006); ^*f*^Cambridge et al. (2011); ^*g*^Doherty et al. (2009); ^*h*^Kono et al. (2015); *Similar growth conditions (Culture medium: DMEM + 10% FPS).

A positive effect on protein J’s relative amplitude was also observed when its gene-specific protein turnover rate was high relative to the bulk protein turnover rate (by setting k_*D, J*_ > k_*D, MEAN*_, Figure 2B). In this case, the increased relative amplitude was a result of reduced daily mean abundance of the modeled protein (and unchanged absolute amplitude), instead of increased absolute amplitude as in Figure 2A. Similarly, a gene-specific turnover rate lower than the bulk degradation rate (k_*D, J*_ < k_*D, MEAN*_) results in increased daily mean abundance and reduced relative amplitude (data not shown). A positive correlation between the daily mean protein synthesis to abundance ratio and the gene-specific protein turnover, as determined by k_*D, J*_ in the model, is consistent with the general definition of protein turnover (turnover rate = synthesis rate/protein abundance, at constant protein abundance). Thus, the model predicts a positive trend between the relative protein amplitude and the gene-specific protein turnover rate, which possibly explains the experimentally observed positive trend between relative protein amplitude and gene-specific turnover (Figure 1C). The model further suggests that high-amplitude proteins are likely to possess a high gene-specific degradation rate relative to the bulk protein degradation rate. Increased absolute amplitude was also observed when the gene-specific degradation rate was actively regulated (time-dependent) and anticorrelated to the synthesis rate (Figure 2C). Thus, particularly high relative amplitudes are possible for a subset of genes even at slow bulk protein turnover, if regulated degradation is fast enough to also reduce the daily mean abundance. Allowing the growth rate to fluctuate according to our experimental data altered the pattern of protein oscillations but did not have a significant effect on the protein amplitude (Figure 2D).

These results demonstrate that the relative amplitude of a protein depends on the bulk protein turnover and the protein’s specific turnover. The bulk protein turnover acts on the protein’s absolute amplitude (and all other proteins), while the latter acts on the daily mean abundance of the protein. The model further shows that the observed reduction in oscillation amplitude of a given protein can be attributed solely to a low bulk protein turnover, corresponding to the experimentally determined growth rate of 0.018 h^–1^ and a bulk protein degradation rate of 0.01–0.05 h^–1^.

## Discussion

Post-transcriptional regulation is an intuitive explanation for the discrepancy between cyclic diurnal transcription and relatively constant protein levels in cyanobacteria. Our transcriptomic, translatomic and proteomic data confirmed this discrepancy and showed that it is not caused by translational regulation. In addition, modeling of the protein response to transcriptional oscillations under biologically relevant parameter settings demonstrated that the experimentally observed decrease in protein oscillation amplitude can be attributed to a slow bulk protein turnover, without the requirement of regulated protein degradation that counteracts transcriptional oscillations. Modeling results further suggested that the bulk protein degradation rate was similar to the daily average growth rate.

The strong correlation between ribosome and mRNA abundance fold changes indicates that protein synthesis oscillates significantly over the day-night cycle and that translation is not regulated between time points (Figure 1A). Synthesis rates were solely based on the ribosome abundance and did not account for within-gene changes in ribosome elongation rate. However, elongation rates were not expected to change significantly on global level between time points, since elongation rates primarily depend on gene-specific properties of the mRNA structure (Riba et al., 2019). Furthermore, variation in elongation rate would more likely result in reduced correlation with mRNA abundance.

In contrast, diurnal protein abundance patterns generally did not show a clear cyclic behavior and did not correlate with protein synthesis oscillations (Figure 1A). Small cyclic patterns were most likely present, but concealed by technical variation and therefore not detectable. As measurement errors were high relative to diurnal changes in protein abundance, the determined median relative protein amplitude of 1.5 was probably overestimated (Figure 1B). The proteome-wide 2.0-fold reduction in amplitude from synthesis to abundance, was comparable to the 2.3-fold reduction determined previously with higher statistical power (Waldbauer et al., 2012). With this approximate ratio taken into account, our model suggests that the bulk degradation rate was in the range of 0.01–0.05 h^–1^, i.e., similar to the daily average growth rate, and in line with degradation rates measured in microalgae and plants (Figure 2A). This was further supported by bulk degradation rates measured in other organisms which are typically in the same magnitude as the growth rate (Table 1). The positive correlation between growth rate and bulk protein degradation has been attributed to a high energy burden of protein turnover when nutrients are limited (Lahtvee et al., 2014).

Our modeling analysis showed that the bulk protein turnover rate (proportional to μ + k_*D, MEAN*_) determines the proteome-wide reduction in amplitude between the synthesis level and the abundance level (mean synthesis:protein amplitude ratio). The model further suggested that gene-specific deviations from the mean synthesis:protein amplitude ratio are determined by deviations in individual protein degradation rates relative to the bulk degradation rate (Figure 2B). Waldbauer et al. (2012) reported variation in the synthesis:protein amplitude ratio (synthesis = mRNA level) across the genome of *Prochlorococcus* MED4 during diurnal growth. While the vast majority of genes in this study also exhibited low amplitude or no oscillations at the protein level, approximately 30 of the 548 analyzed proteins showed an amplitude fold change greater than 2. However, the relatively high amplitude of these proteins was not caused by particularly strong oscillations in protein synthesis relative to other genes. Instead, protein synthesis oscillations of these genes appeared to be less dampened at the level of protein relative to other genes, as indicated by a lower synthesis:protein amplitude ratio. Our model suggests that such outlier proteins are subjected to a high gene-specific degradation rate (i.e., gene-specific protein turnover), which increases the relative amplitude of oscillations by reducing the protein’s daily mean abundance without affecting the absolute amplitude. This was further indicated in our experimental data (Figure 1C), where a positive trend between the relative protein amplitude and gene-specific protein turnover (daily mean synthesis rate/daily mean abundance) was detected. A degradation rate for a given protein that is 10-fold higher than the bulk degradation rate is not unrealistic, as gene-specific degradation rates were shown to span two to three orders of magnitude in *Lactococcus lactis* (Lahtvee et al., 2014). Furthermore, artificially increasing degradation rate, by fusing a ssrA degradation peptide, increased the relative amplitude and decreased the phase shift of a diurnally expressed yellow fluorescent protein in *Synechococcus elongatus* PCC 7942 (Chabot et al., 2007).

The protein oscillation model assumes a constant cellular protein concentration. This assumption was largely satisfied over the day-night cycle, according to measurements of total protein content in cell extracts. The assumption of a constant cellular protein concentration constrains bulk protein synthesis to be proportional to the sum of bulk protein degradation and growth dilution. Consequently, a decreasing protein concentration during night time will lead to an overestimated bulk protein synthesis rate by the model. This will in turn result in an overestimated rate change of each protein’s (J) concentration during night time. However, as the cellular protein concentration increases to its original level during sunrise, the opposite effect will occur. That means bulk protein synthesis will be underestimated and the rate change of each protein’s concentration will be underestimated, which compensates for the overestimated rate change during the night. Thus, small changes in cellular protein concentration will not change the simulated protein amplitude significantly, but rather alter the diurnal pattern of protein abundance. This is analogous to the effect of setting a constant growth rate vs. setting a fluctuating growth rate (Figure 2D).

Cyclic transcription has been shown to peak near time points of the day-night cycle when the corresponding function is expected to be needed by the cell (Waldbauer et al., 2012; Beck et al., 2014; Saha et al., 2016; Strenkert et al., 2019). However, the regulatory purpose of a diurnally shifting transcriptome appears less meaningful, since the impact on the functional protein level is significantly diminished. It is nonetheless possible that well-timed, yet small, changes in protein abundance results in a growth benefit that increases survival fitness in a natural environment. Furthermore, our model shows that these changes would become increasingly relevant in a condition that permits higher growth rates, such as an eutrophicated lake exposed to intense sunlight (Figure 2A, right). Indeed, *Synechocystis* can grow with a growth rate as high as 0.16 h^–1^ (van Alphen et al., 2018). This growth rate would correspond to a daily average protein turnover (μ + k_*D, MEAN*_) of approximately 0.12 h^–1^, considering a diurnal growth pattern and that the bulk degradation rate is typically similar and dependent on the growth rate (Table 1). Protein levels in cyanobacteria do change significantly in response to changes in light intensity, if allowed to adjust to a steady state (Jahn et al., 2018). Yet, during diurnal growth, the co-occurrence of a largely constant proteome and considerable metabolic shifts suggests that allosteric interactions play an important regulatory role. For example, CO_2_ fixation is inactivated during the night through an allosteric mechanism where the regulatory protein CP-12 binds and inactivates the Calvin cycle enzymes phosphoribulokinase and glyceraldehyde-3-phosphate dehydrogenase (Tamoi et al., 2005). Glycogen degradation is another potential target of allosteric regulation since it mostly occurs during the night, even though the abundance of glycogen phosphorylase does not change over the day-night cycle (Supplementary Table 1).

Our results also have implications for synthetic biology in cyanobacteria. There have been many efforts to control the abundance of heterologous proteins in *Synechocystis*, at both the level of translation, through alteration of RBS sequence (Thiel et al., 2018), and at the level of degradation, through a synthetic ssrA peptide with a calculated homology to the native sequence (Landry et al., 2013). The perceived ribosome binding site affinity is not an accurate predictor of protein levels, even when comparing ribosome binding sites with the same heterologous protein (Thiel et al., 2018). It is possible that ribosome profiling, which provides a measure of ribosome occupancy across the entire transcript, could provide insight as to how genetic context affects translation of heterologous proteins. The findings in this study suggest that faster changes in a heterologously expressed protein’s abundance can be achieved, if its synthesis rate and degradation rate is high, i.e., if its gene-specific protein turnover is high. In case transcription of the heterologous gene is from a promoter that has an inherent oscillation, then an increased degradation rate, through e.g., a strong degradation tag, could increase oscillations in the protein level. At the same time, a slow bulk protein turnover will extend the time needed for that protein to reach its steady-state abundance, since the cellular protein space is limited. This appears to be the case in cyanobacteria cultures grown at constant light. In a study on the induction kinetics of YFP from various promoters in *Synechocystis*, the protein accumulated for five days after induction with rhamnose before reaching a steady state (Behle et al., 2020). In day/night cultivations, the change in the target’s protein abundance will be slower still, as total transcription and/or translation is globally downregulated at night, by inactivation of RNA polymerases and/or ribosomes (Hood et al., 2016). Therefore, comparisons of gene expression constructs, such as promoters or ribosome binding sites, should occur only after steady-state has been reached.

In conclusion, we show that the relatively constant proteome during diurnal growth can be explained by low protein turnover. A relatively high bulk protein turnover is required to obtain significant diurnal changes at the global proteome level. To minimize protein turnover energy costs and improve fitness under growth limited conditions, cyanobacteria may instead have evolved allosteric mechanisms to regulate metabolic shifts. Such adaptation may be particularly relevant for photosynthetic organisms as their energy supply is limited to times of the day with sunlight exposure. Identifying potential allosteric regulation of key enzymes in cyanobacteria could assist future metabolic engineering attempts to accelerate carbon fixation or divert metabolic flux, as these enzymes could become targets for protein engineering. Incorporating allosteric regulation into metabolic models would also improve their prediction capability when simulating genetic knockouts that result in altered metabolic flux patterns. Furthermore, our results suggest that changes in transcription or translation are not necessarily a good predictor of diurnal changes in enzyme concentration, or metabolic flux.

## Data Availability Statement

The datasets presented in this study can be found in online repositories. The names of the repository/repositories and accession number(s) can be found below: ProteomeXchange dataset PXD023812, https://www.ncbi.nlm.nih.gov/bioproject/PRJEB42778.

## Author Contributions

EH and JK conceived and designed the experiments. DV, JK, and MJ performed the experiments. JA-S, JK, and MJ conducted the computational data analysis. All authors contributed to the manuscript.

## Conflict of Interest

The authors declare that the research was conducted in the absence of any commercial or financial relationships that could be construed as a potential conflict of interest.

## Footnotes

1 https://github.com/J-Karlsen/diurnal-gene-expression

2 http://www.ebi.ac.uk/pride

3 https://github.com/Asplund-Samuelsson/ribopipe

4 https://github.com/fhcrc/seqmagick
