# Supplementary material for: Slow Protein Turnover Explains Limited Protein-Level Response to Diurnal Transcriptional Oscillations in Cyanobacteria
Source: Front Microbiol. 2021 Apr 14;12:657379. doi: 10.3389/fmicb.2021.657379 (PMC8237939; doi:10.3389/fmicb.2021.657379)
Supplement: Supplementary file 1 [file Data_Sheet_1.pdf]

Supplementary Material

1 Supplementary Figures

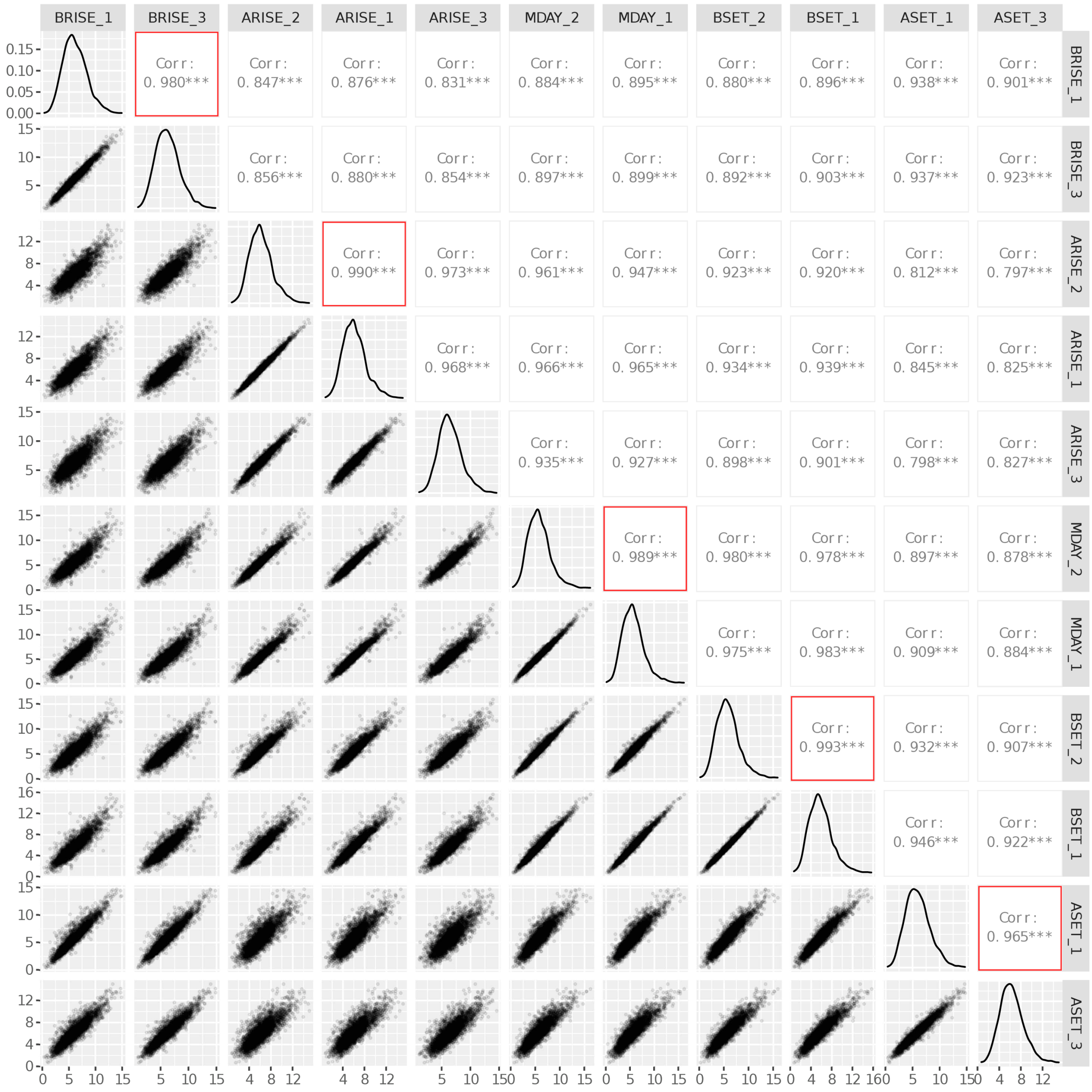

Supplementary Figure 1. The correlation between replicate ribosome profiling samples collected from different cultivations is similar to the correlation between replicate samples collected from the same cultivation. The figure displays all possible combinations of correlations between the eleven ribosome samples collected from cultivation 1 and 2. Points in each scatter plot show the ribosome abundance (log_2RPKM) of individual genes. Corresponding Pearson's correlation coefficients are shown above the diagonal (log_2RPKM distributions), and comparisons between replicates are highlighted in red. BRISE = 1 h before sunrise, ARISE = 1 h after sunrise, MDAY = midday, BSET = 1 h before sunset, ASET = 1 h after sunset. The digit in each sample label is the replicate ID; "1" and "2" were sampled in cultivation 1 (chronological order of sampling), and "3" was sampled in cultivation 2.

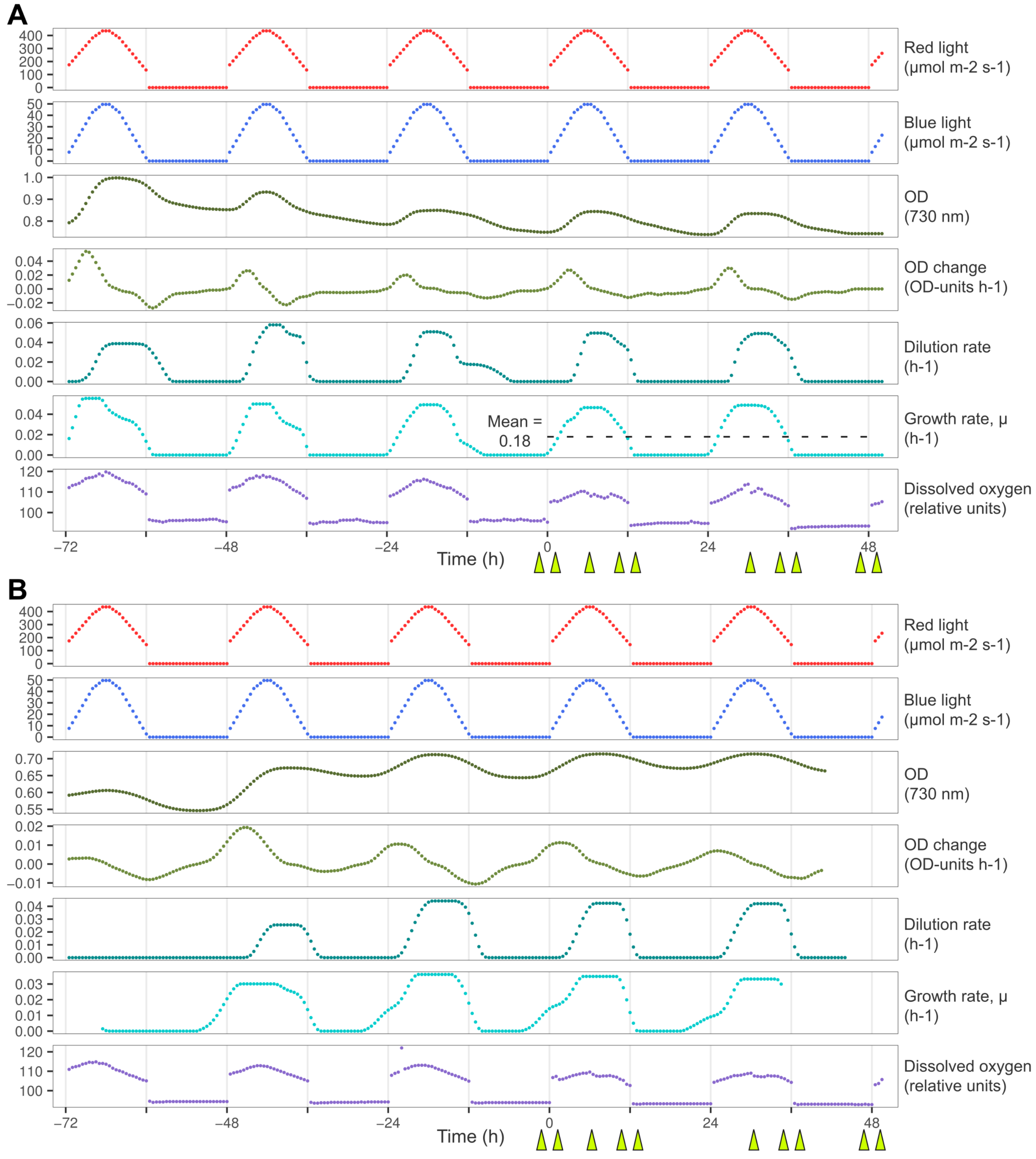

Supplementary Figure 2. The diurnal growth rate pattern correlates with light intensity with a daily average of 0.18 h^{-1}. Panel A and B shows data from cultivation 1 and 2, respectively. Data is shown for time points three days before sampling (-72 to 0 h), and for the two days when sampling was conducted for RNA sequencing, ribosome profiling and LC-MS-MS (0-48 h, green triangles). The light intensity of blue and red light was set according to a sinusoidal function during the day, and zero during night. OD_{730}, OD_{730} changes, dilution rates and growth rates were filtered from noise by calculating the (truncated) average of neighboring data points. The daily average growth rate was calculated over two days (0-48 h) in cultivation experiment 1 (black dash line). Growth rates estimated in cultivation 2 were prone to error because of relatively high noise levels and low data

acquisition rate (1-min intervals, compared to 30-s intervals in cultivation 1). The amount of dissolved oxygen in the culture medium serves as a proxy for photosynthetic activity and growth rate (a value = 100 corresponds to the saturated oxygen concentration under atmospheric oxygen partial pressure). The number of data points were reduced to two observations per hour before plotting.

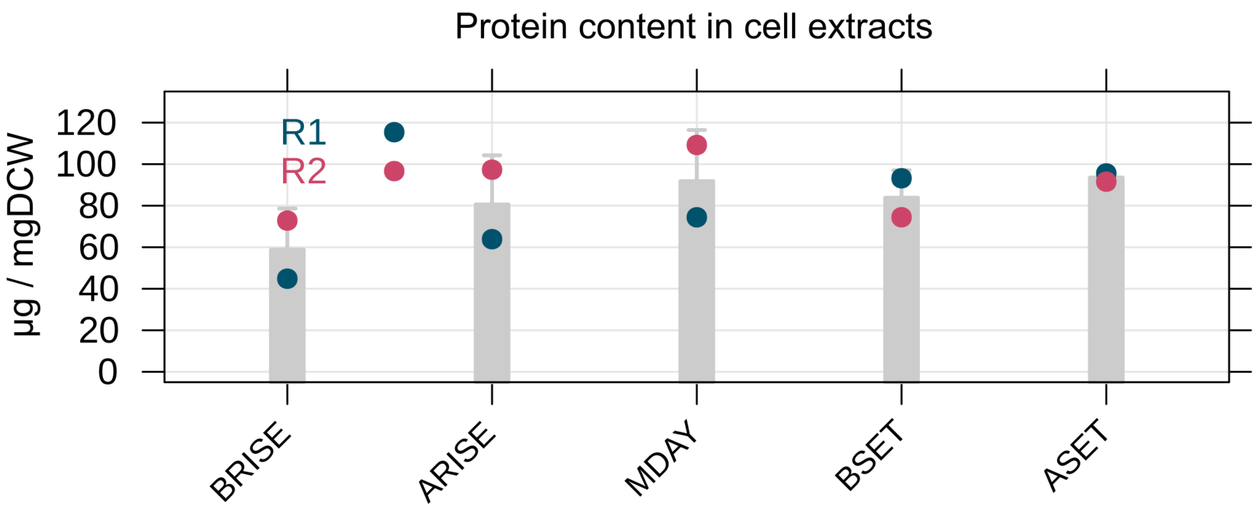

Supplementary Figure 3. The total protein content in cell extracts did not change significantly across the day-night cycle. The height of bars shows the average total protein content in cell extracts (normalized to dry cell weight, DCW) at each time point, determined from two replicate measurements (R1 and R2). BRISE = 1 h before sunrise, ARISE = 1 h after sunrise, MDAY = midday, BSET = 1 h before sunset, ASET = 1 h after sunset. Differences between time points were not statistically significant ($p > 0.4$).

2 Supplementary Tables

See file: "TableS1_summary_statistics_RNAseq_RP_MS.xlsx"

Supplementary Table 1. Summary statistics for RNA sequencing, Ribosome profiling and Protein MS. BRISE = 1 h before sunrise, ARISE = 1 h after sunrise, MDAY = midday, BSET = 1 h before sunset, ASET = 1 h after sunset. P-values (p_val) were determined using one-way ANOVA, and were subsequently adjusted for multiple hypothesis testing using the Benjamini-Hochberg/FDR method (q). A diurnal change in mRNA/protein abundance was considered significant if $q < 0.1$.
